# Cross-Linking of Oxidized Hydroxypropyl Cellulose in Paper: Influence of Molecular Weight and Polymer Distribution on Paper Wet Strength Development

**DOI:** 10.3390/gels9030206

**Published:** 2023-03-09

**Authors:** David Seelinger, Markus Biesalski

**Affiliations:** Ernst-Berl-Institut Macromolecular and Paper Chemistry, Technical University Darmstadt, Alarich-Weiss-Str. 8, 64287 Darmstadt, Germany; david.seelinger@tu-darmstadt.de

**Keywords:** hydroxypropyl cellulose, wet tensile strength, wet strength resin, polymer degradation, polymer distribution in paper

## Abstract

With the overarching aim for the development of sustainable, nontoxic wet strength agents for paper, a novel polymer gel system based on oxidized hydroxypropyl cellulose (keto-HPC) cross-linked with polyamines was investigated in detail to gain a deeper insight into the wet strength mechanism. When applied to paper, this wet strength system significantly increases the relative wet strength by using only low amounts of polymer, and it is therefore comparable with established wet strength agents based on fossil resources, such as polyamidoamine epichlorohydrin resins. With the help of ultrasonic treatment, keto-HPC was degraded with respect to its molecular weight and further cross-linked in paper using polymeric amine-reactive counterparts. The resulting polymer-cross-linked paper mechanical properties were analyzed with respect to the dry and wet tensile strength, respectively. In addition, we analyzed the polymer distribution using fluorescence confocal laser scanning microscopy (CLSM). If high-molecular-weight samples are being used for cross-linking, we do find accumulation of the polymer mainly on the surface of the fibers and at fiber crossing points, accompanied with enhancing strong effects on paper’s wet tensile strength. In contrast, if low-molecular-weight (i.e., degraded) keto-HPC is being applied, the macromolecules are capable of entering the inner porous structure of the paper fibers, and almost no accumulation at the fiber crossing points is observed, which also results in a lowered wet paper tensile strength, respectively. This insight into wet strength mechanisms of the keto-HPC/polyamine system can thus lead to new opportunities for the development of alternative biobased wet strength agents where molecular weight dependence of the wet tensile properties allows for a fine tuning of mechanical properties in the wet state.

## 1. Introduction

Paper and paper-based products have become an essential part of our everyday life. Due to the increasing importance of high-performance papers, high demands are often made on their properties. For example, paper used for packaging can be equipped with barrier properties against oxygen [1], water vapor [2] or fats [3], and also achieves high dry and wet tensile strength. The latter is crucial for paper products that come in contact with water, such as tissue paper, bottle labels and many more [4]. In general, paper is made from a network of cellulose fibers that bond to each other by different mechanisms, including (but not limited to) hydrogen bonding, Van der Waals forces, capillary forces and interdiffusion. Many of these bonding mechanisms are highly sensitive to water and summarized in numerous reviews by experts [5,6,7,8]. Consequently, when paper is wetted, only a small fraction (less than 5%) of the initial dry tensile strength remains [5]. To ensure that these paper products meet the desired mechanical stability in the wet state, the paper fibers must first be reinforced. Additives that are able to reinforce the cellulose fiber network are known as wet strength agents.

Over the last decades, the rising demand for paper and paper-based products has led to research and development of very cost-efficient wet strength additives such as melamine- and urea-formaldehyde [9,10], polyvinylamine [11] and polyamidoamine epichlorohydrin (PAAE) [12]. These wet-strengthening agents act by homo- and co-cross-linking with themselves and with the lignocellulosic fibers and forming polymer networks, i.e., gel-like structures. The current market-dominating wet strength agent, PAAE, was already developed in the 1960s and is at the moment in its fourth generation [13]. Despite the good wet strength and cost efficiency, PAAE wet strength agents have a serious disadvantage: the resin contains harmful by-products—mainly 1,3-dichloropropane (DCP) and 3-monochloropropane-1,3-diol [14]. These by-products are formed by the hydrolysis of epichlorohydrin and are classified as potentially carcinogenic. This limits the application of epichlorohydrin-based wet strength agents, and the regulations of these harmful residues in PAAE have been continuously tightened, which is why PAAE wet strength agents must be improved. Particularly in the food packaging industry, DCP and other adsorbable organic halogenides (AOX) are harmful, and PAAEs are therefore restricted. In this field of application, the PAAE resins need to be purified from DCP and AOX first, using a complicated nanofiltration process [15] or biodehalogenation via special microbes [16], until the AOX concentration is below a certain detection limit. Therefore, AOX residues pose a potential risk of further regulations or even a complete ban on epichlorohydrin-based wet strength agents in the future. Consequently, there is a genuine need for the development of new wet strength agents. Alternative approaches aim at novel cross-linking methods (e.g., photochemistry [17]) and the development of nontoxic systems based on biogenic starting materials. Cellulose is an interesting candidate for the development of such a nontoxic wet strength system [18,19], because it provides a number of valuable advantages: it is the most abundant biopolymer in nature and is therefore highly available in bulk; it is also nontoxic and provides a high number of functional groups available for modification. However, the poor solubility of cellulose in common organic solvents such as alcohols, tetrahydrofuran or chloroform limits its potential. [20] To overcome this problem, researchers use cellulose derivatives such as hydroxypropyl cellulose (HPC) as starting material for further modifications such as esterification [21]. HPC at present is highly available because of its use in the food and pharma industry [22,23]. This cellulose ether is produced by a ring-opening addition of propylene oxide and alkali-treated cellulose, and therefore provides, similarly to cellulose, three hydroxy groups per anhydroglucose unit for further modification. However, these introduced hydroxy functions can undergo repeated etherification, and therefore HPC reaches a degree of substitution (DS) above three. Here, the DS is defined as molar substitution (MS) [24].

**Figure 1:**
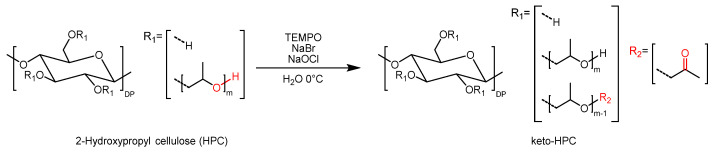
Introduction of carbonyl functions into HPC by TEMPO-mediated oxidation.

For the generation of a wet strength agent made from HPC, covalent cross-linking points are necessary, and therefore so is the introduction of reactive groups. In this respect, we described in our previous work the modification of HPC via TEMPO (2.2.6.6-tetramethylpiperidinyl-1-oxyl)-mediated oxidation. During this procedure, the 2-hydroxypropyl side chains of HPC are transformed to terminal ketone moieties (Figure 1). In a recent study, oxidized HPC (keto-HPC) was used to enhance the wet tensile strength of paper by using low-molar-mass diamine (hexamethylene diamine) as a model co-cross-linker agent [25]. In first concepts, the focus of these initial studies was on understanding and controlling the oxidation kinetics in the preparation of keto-HPC as well as first conceptual investigations of the possibility to form polymer gel structures in bulk. The latter conceptually is similar to the formation of CNC/CNF aerogels described recently in the literature [26]. Our first experiments could also show the beneficial improvement of keto-HPC cross-linked with polyamines. Open questions that were left at that point and on which we are following-up in the present report address the use of different polymeric amines as co-cross-linkers, as well as understanding the influence of the molecular characteristics of keto-HPC and polyamine; in particular, the molecular weight of the polymer on the final impact on the mechanical properties of paper in the wet state.

**Figure 2:**
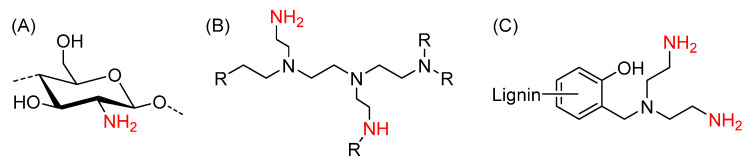
Polyamines used in this work for the cross-linking of keto-HPC: (**A**) chitosan, (**B**) (branched) PEI, and (**C**) Kraft lignin diethylamine (KL-DETA).

Hence, in the present work, we focus on a more detailed investigation of the molecular weight influence on this novel wet strength system made from keto-HPC cross-linked with different polyamines. Therefore, HPC with a high molecular weight was used as a starting material for the TEMPO-mediated oxidation and successively cross-linked with high-molecular-weight polyamines instead of low-molar-mass diamines. As we describe in our previous work, amines are suitable cross-linkers for keto-HPC due to the formation of covalent bonds, e.g., enamines and imines. These covalent bonds are known for their reversibility, and could therefore also enhance the recyclability of wet strengthening paper [19]. Polyamines are particularly interesting for the development of wet strength agents based on keto-HPC as they have a high amine content and therefore increased cross-linking possibility, which enables interfiber cross-linking. In the present manuscript, we investigated the wet strengthening effect in paper of keto-HPC cross-linked with polyamines such as polyethylenimine, aminated Kraft lignin and chitosan, respectively. These polyamines (shown in Figure 2) offer several advantages, e.g., chitosan is obtained by deacetylation of chitin from crustaceans and is biodegradable in the environment while also providing antibacterial properties [27,28,29].

The second multifunctional amine we are utilizing as a wet strength agent for paper in combination with keto-HPC is an aminated Kraft lignin derivative. Lignin is generated as a by-product in the pulp and paper industry, and due to its unique chemical structure provides great potential for many applications [30,31]. At present, however, the majority of lignin is used as fuel for energy generation. Therefore, it is highly cost-efficient with good availability. In order to utilize lignin in combination with keto-HPC as a wet strength agent, we modified Kraft lignin via Mannich reaction, as this reaction is already well described in the literature [32]. As the third and last polyamine, we study in detail the strengthening properties in paper of keto-HPC in combination with branched polyethylene imine, which is commercially synthesized by the cationic ring-opening polymerization of aziridine, and is therefore available in bulk [33]. Note that although both chitosan and aminated lignin are of biogenic origin and thus would add a higher degree of sustainability, PEI is an already-established retention and drainage aid for paper making, and also provides good fixation effects when applied as anionic trash-fixing agent [34]. As such, it can serve as a model polyamine in an excellent manner.

To investigate the molecular weight influence, ultrasonic treatment was implemented as a nonchemical method to reduce the chain length of keto-HPC and measure the wet strength impact afterwards. Wet strength in paper is expected to decline with reducing molecular weight, as proposed by Li et al. [35]. Furthermore, our previous results have shown that for wet strength papers, a high polymer dosage of keto-HPC (4 wt% based on used fibers) is necessary [25], which limits possible transfer into an industrial application of keto-HPC as a wet strength agent. In order to overcome this limitation reported in our previous study, we now choose a combination of high-molecular-weight keto-HPC and high-molecular-weight polyamines to enhance the wet strength performance in paper by cross-linking and the formation of a polymeric gel. With this, we follow the hypothesis that the use of higher-molecular-weight precursor agents should increase the probability of bridging larger gaps between fibers while forming interfiber cross-links. The latter should thus result in a higher wet-strengthening effect with the aim of reducing the necessary high amounts of wet strength agent keto-HPC required for the generation of wet strength paper. Finally, we analyze the polymer distribution of keto-HPC in paper using confocal laser scanning microscopy to determine areas where keto-HPC reinforces the paper and gain more insights into the wet-strengthening mechanism of keto-HPC in general.

## 2. Results and Discussion

### 2.1. Hydrogels Made from Keto-HPC Cross-Linked with Polyamines

In this study we investigated in detail the reinforcing effect of cellulosic hydrogels made from cross-linked keto-HPC on lab-made paper from cotton linter fibers. In order to accomplish this, the first step is the oxidation of HPC via TEMPO-bleach protocol, as recently described in previous work. During this procedure, the secondary hydroxy groups of the HPC ether side chain are converted into carbonyl functions, as can be inferred from Figure 1. The latter was confirmed via several spectroscopic methods such as attenuated total reflection infrared spectroscopy (ATR-IR) and NMR methods, respectively. A carbonyl-specific IR absorbance band at 1723 cm^−1^ is observed, which proves the chemical identity of native HPC. The conversion of the secondary hydroxy group to carbonyl was in addition confirmed by ^1^H-NMR, HSQC-NMR (heteronuclear single quantum coherence) and COSY-NMR (correlation spectroscopy), respectively [25]. This conversion results in an HPC derivative with a higher reactivity, for instance, towards amines. The latter enables reactions such as *Schiff*-base formation, which can be used for the covalent and reversible cross-linking of keto-HPC. The oxidation is carried out with an excess of sodium hypochlorite in the presence of TEMPO and sodium bromide in water at mild conditions (0 °C, pH 10), as described in the experimental Section 4.2. These mild conditions are necessary for avoiding significant polymer degradation [25]. The amount of sodium hypochlorite plays a predominant role in allowing control of the reaction, and thus control of the degree of oxidation. For keto-HPC synthesis, a DOx of 1.5 was selected, which means that ~68% of the secondary hydroxy groups are oxidized to keto groups. This DOx enables high reactivity towards amine cross-linkers while preventing high loss of molecular weight due to unwanted side reactions. The resulting DOx of 1.50 was determined by ^1^H-NMR spectroscopy (see Figure S1). Compared to our previous work, HPC with a very high molecular weight of M_w_ = 6.5 × 10^5^ g mol^−1^ was used as starting material for the TEMPO-mediated oxidation. As described below, variation in the molecular weight of the keto-HPC was further achieved by ultrasonic treatment. The latter allows for a thorough investigation of the molecular weight influence on wet strength in paper. Secondly, with the use of high-molecular-weight HPC polymers the probability of interfiber cross-linking in paper is theoretically increased due to an increased polymer chain length being more efficient in bridging larger gaps between individual fibers.

**Figure 3:**
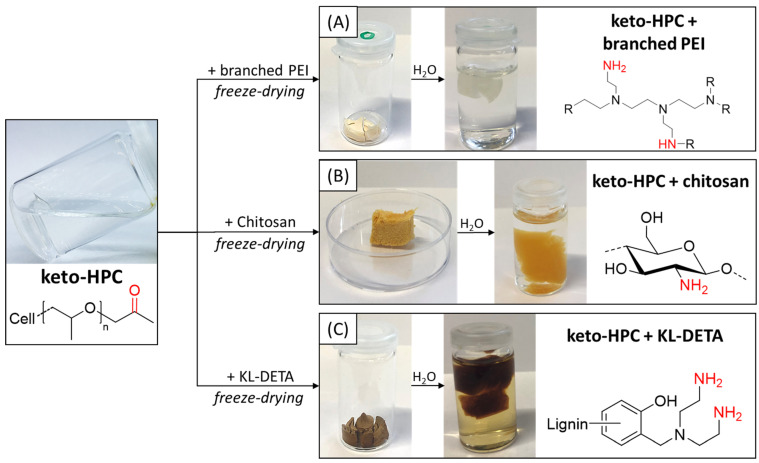
Hydrogels made from keto-HPC (**A**) with PEI (**B**) with chitosan and (**C**) KL-DETA.

Keto-HPC cross-links with primary amines by imine formation, which was confirmed by infrared spectroscopy (IR) and is discussed elsewhere [25,36]. As such, it is possible to cross-link keto-HPC with polyamines such as chitosan, PEI and aminated Kraft lignin to form stable hydrogels. In addition, amide formation between carboxylic acid moieties present by oxidation of C6-OH groups and primary amines can occur in principle. First, we investigated hydrogel formation in bulk, i.e., without applying the reactive polymers to paper. For the formation of such hydrogels, keto-HPC was dissolved in distilled water to result in a 4 wt% solution, and it was cross-linked with the respective polyamine by the addition of PEI, chitosan or KL-DETA. To achieve the highest possible cross-linking and to avoid problems arising from high viscosities, a carbonyl-to-amine ratio of 0.25 for PEI and 1.0 for KL-DETA and chitosan was adjusted. After freeze-drying of this mixture, a porous polymer network was collected as described in the experimental Section 4.3. The resulting freeze-dried polymer network was immersed in distilled water and was stable in water for several days, as shown in Figure 3. Note that the supernatant liquid of the hydrogel made from keto-HPC and KL-DETA shows discoloration due to dissolution of unbound KL-DETA. However, hydrogels made from keto-HPC cross-linked with KL-DETA were nevertheless stable in water for several days and discoloration stopped after approximately 18 h. It can also be observed that the freeze-dried polymer networks swelled in water (Figure 3). Within this work, however, the degree of swelling of the formed model hydrogels were not determined, as a direct comparison to those gels formed within paper is not trivial.

### 2.2. Cross-Linking of Keto-HPC with High-Molecular-Weight Polyamine in Paper and Impact on Wet Tensile Strength

As the general applicability of the polymers to cross-link into polymeric gel-structures was proven on a qualitative base, in the next step, we investigated the application of keto-HPC cross-linked with different polyamines inside paper samples in detail. Lab-made paper of cotton linter fibers with a grammage of 100 g m^−2^ was prepared and impregnated with the respective polymer solutions via size press coating. The combination of keto-HPC and the respective polyamine was applied as an aqueous solution with a relative coating weight of 1.5 wt% based on dry fibers, as described in experimental Section 4.3, Section 4.4 and Section 4.5. Similar to the model reactions, the molar ratio of carbonyl to amines was chosen to be 1 for chitosan and KL-DETA, respectively. Because of the higher amine concentration with PEI polymer (8.74 mmol/g compared to chitosan: 6.2 mmol/g and KL-DETA: 1.75 mmol/g), a lower molar ratio of 0.25 was chosen to avoid inhomogeneous coatings due to high viscosities. The cross-linking of keto-HPC in paper with the respective polyamine took place at norm-climate condition during drying (23 °C, 50% RH). After drying, tensile strengths were measured both in dry and wet state using DIN protocols.

**Figure 4:**
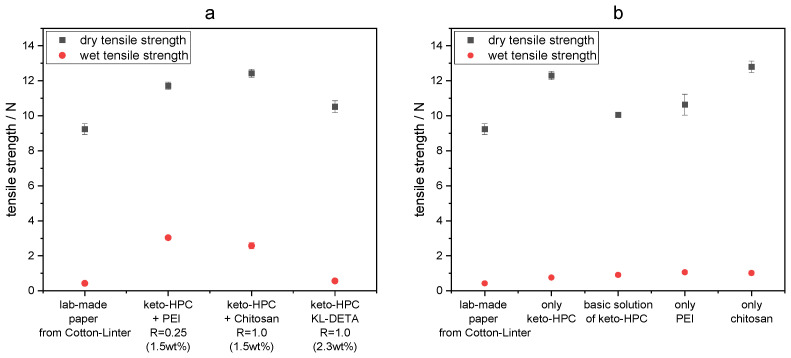
(**a**) Dry (patterned black) and wet (red) tensile strength of cotton linter paper coated with keto-HPC DOx1.5 in combination with PEI, KL-DETA and PEI, respectively. (**b**) Dry (patterned black) and wet (red) tensile strength of cotton linter paper coated only with keto-HPC DOx1.5, PEI, chitosan or KL-DETA as reference measurements. Untreated lab-made paper of cotton linter fibers with a grammage of 100 g m^−2^ was used as untreated paper reference.

As Figure 4a shows, lab-made papers of cotton linters utilized with keto-HPC in combination with PEI and chitosan, respectively, achieve high wet tensile strength values of 3.0 N and 2.6 N, respectively, compared to untreated paper samples (0.43 N). Note that keto-HPC and chitosan alone showed no increase in wet strength compared to untreated paper (Figure 4b). However, PEI is known for its moderate wet-strengthening effect in paper, but as shown in Figure 4b, the wet tensile strength of paper samples treated with PEI alone increases only slightly [37]. Here, the applied quantity of PEI in the paper sample is based on the amount used to cross-link keto-HPC. Interestingly, when keto-HPC is applied to paper by an aqueous coating with a basic pH of 9 (adjusted with NaOH), acetal bonds are formed after drying, which also results in moderate wet strength values. The chemical identity of the newly formed acetal bonds can be proven by corresponding signals in IR-spectra, as shown in Figure S2. These results suggest that that neither PEI nor keto-HPC alone is the main contributor to wet strength, but rather the combination of keto-HPC and PEI leads to high wet tensile strength.

**Figure 5:**
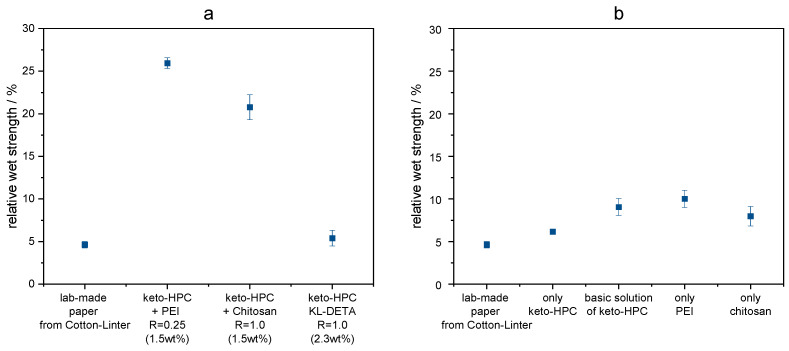
(**a**) Resulting relative wet strength of lab-made paper samples of cotton linters treated with keto-HPC in combination with PEI, chitosan and KL-DETA, respectively. (**b**) Resulting relative wet strength of lab-made paper samples.

Unlike established wet strength systems, keto-HPC in combination with polyamines only slightly increases the dry strength. The latter yields exceptionally high relative wet strength values of 20% and >25%, respectively, of paper samples utilized with keto-HPC in combination with PEI and chitosan, respectively, with a coating weight of only 1.5 wt% (relative to the mass of dry cellulose fibers), as shown in Figure 5a. This finding is particularly interesting since PEI, being cost-efficient and already available in bulk quantities, and chitosan as a biogenic cross-linker, could form—in combination with keto-HPC—an almost fully biogenic wet strength system that has not yet been reported to the best of our knowledge. Both chitosan and PEI are therefore suitable cross-linkers for keto-HPC while enhancing wet strength, even with low amounts of polymer applied to paper samples.

As mentioned before, our previous work could already show the beneficial wet strength improvement of keto-HPC cross-linked with polyamines in paper. However, by using much higher-molecular-weight samples of keto-HPC in combination with polyamines in the present study, we are capable of increasing the efficiency of our wet strength agent keto-HPC compared to our previous work by a factor of 300%. Hence, only about a third of the polymer amount (26.0% rel. WS with 1.5 wt% coating weight) is necessary to generate similar wet strength values if compared to previous investigations (29.0% rel. WS with 4.0 wt%) [25]. Note that chitosan itself has been described before to enhance wet strength in paper in the literature; however, the described systems rely on a more sophisticated cross-linking system, for example, glyoxal/Zn(NO_3_), maleic anhydride or polyhexamethylene guanidine [38,39,40].

When keto-HPC is cross-linked with KL-DETA in paper, relative wet strength values of only about ~7−8% are achieved (see Figure 5a). The reason for such low values of the relative wet strength in comparison to chitosan or PEI can be explained by a low concentration of amine groups within KL-DETA of only 1.75 mmol/g. In comparison, the used chitosan amine group content was 6.2 mmol/g and the same for branched PEI was 8.74 mmol/g, respectively. This reduces the probability of high conversion of carbonyl functions to react with amines, and therefore decreases cross-linking density in the final polymeric gels, respectively. The latter also explains the insufficient cross-linking and leaching of unreacted lignin molecules from the gels, as discussed above. In order to enhance the cross-linking efficiency of the amine-functional lignin, and thus to further transfer such biomacromolecules into valuable wet strength additives, further adjustment of the synthetic pathways to KL-DETA is necessary. The latter, however, is beyond the scope of the present studies, and following investigations were performed with chitosan and PEI only.

**Figure 6:**
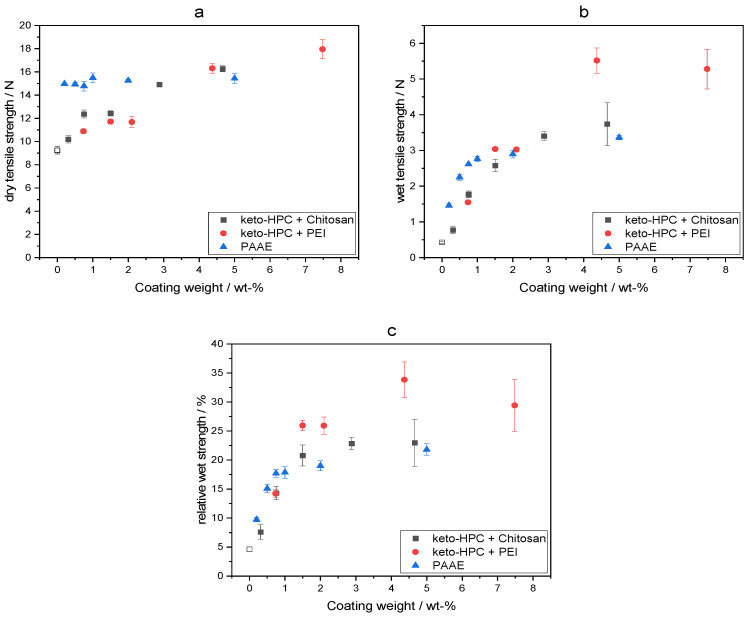
Influence of the coating weight of keto-HPC in combination with chitosan (black), PEI (red) and PAAE (blue), respectively, based on dry cotton linter fibers on dry tensile strength (**a**), wet tensile strength (**b**) and relative wet strength (**c**).

As a next step, we were interested in learning about the influence of the concentration of the added keto-HPC/polyamine systems to achieve a minimum of 15% relative wet strength. The latter is the industry threshold for a “wet strengthened paper sheet”. For this, we again investigated the resulting dry and wet tensile strength of lab-made paper samples with increasing polymer amount as shown in Figure 6a–c. It can be observed that only 0.75 wt% of keto-HPC relative to the fiber weight, in combination with chitosan and PEI, respectively, is necessary to generate a relative wet strength of almost 15%. We compared these results with commercially available wet strength additives (superimposed date can be found in Figure 6 as well), such the polymeric wet strength additive PAAE. Here, very similar values were observed for the relative wet strength of our model paper.

PAAE was added to cotton linter fibers during the paper making process to mimic the industrial wet-end application (For detailed description of paper preparation with PAAE see Supplementary Materials, Section S6). As shown in Figure 6a–c, coating weights above 3.0 wt% does not result in higher relative wet strength for the keto-HPC + polyamine wet strength system. As expected, the relative wet strength of paper with PAAE only increases slightly above a coating weight of 1 wt% due to the decreasing retention of PAAE to fibers during the paper-making process [41].

**Figure 7:**
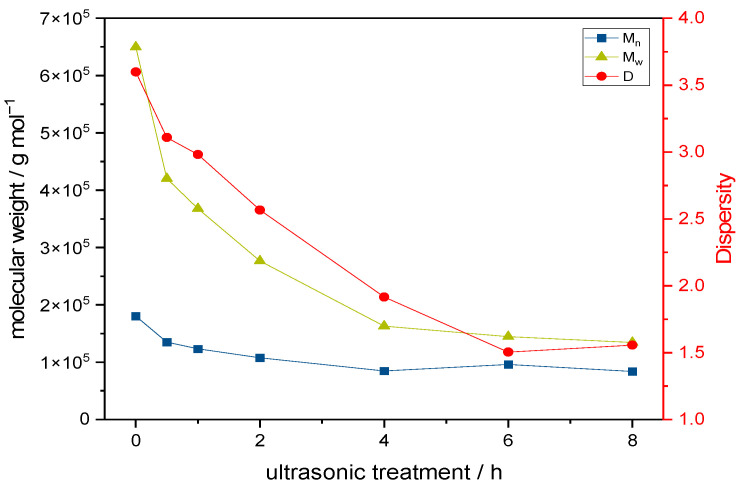
Polymer degradation and polymer weight distribution of keto-HPC DOx1.5 via ultrasonic treatment.

Next, we were interested to further investigate the influence of the molecular weight of keto-HPC on wet strength in paper. In a first step here, we degraded keto-HPC polymer chains to adjust the molecular weight. Ultrasonic treatment is a suitable method for the controlled polymer degradation of cellulose ethers such as keto-HPC [42]. This method of polymer degradation comes with several advantages compared to chemical or thermal degradation processes, such as the avoidance of chemical alternation of the degraded polymer. The keto-HPC polymer chains were degraded via ultrasonic treatment in aqueous solution, and the molecular weight distribution was followed by size exclusion chromatography (SEC). The weight average molecular weight of keto-HPC decreases from 6.5 × 10^5^ g mol^−1^ to approximately 1.3 × 10^5^ g mol^−1^ during an ultrasonic treatment of 8 h, and molar mass reduction levels off at this point in time (Figure 7). Similar to other groups, we observed that the polymer dispersity decreases with decreasing molecular weight because of larger polymer chains degrading faster [42]. It should be noted that during the synthesis of keto-HPC, there is already some decrease in molar mass (native HPC: M_w_ = 9.6 × 10^5^ g mol^−1^; after oxidation: M_w_ = 6.5 × 10^5^ g mol^−1^). After 4 h of ultrasonic treatment, ^1^H-NMR spectroscopy revealed no changes in DOx (See Figure S3 in Supplementary Materials). Since there is no chemical alternation of keto-HPC, the molar ratio of keto-HPC and PEI is the same as for undegraded keto-HPC + PEI, and therefore allows for the investigation of degraded keto-HPC on the resulting wet strength in paper.

**Figure 8:**
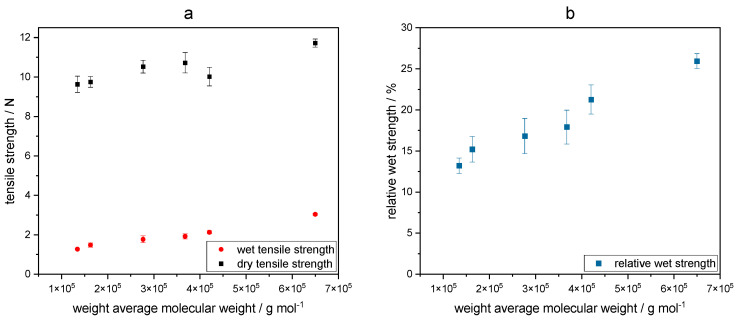
Influence of the weight average molecular weight of keto-HPC DOx1.5 in combination with PEI on dry and wet tensile strength (**a**) and relative wet strength (**b**) applied to cotton linter paper with a polymer amount of 1.5 wt%.

As shown in Figure 8, the wet strength increases with increasing molecular weight of keto-HPC from 1.27 N to 3.04 N. The relative wet strength increases at the same time from about 12% at a molecular weight of 10^5^ g/mol to more than 25% at a molecular weight of about 10^6^ g/mol.

**Figure 9:**
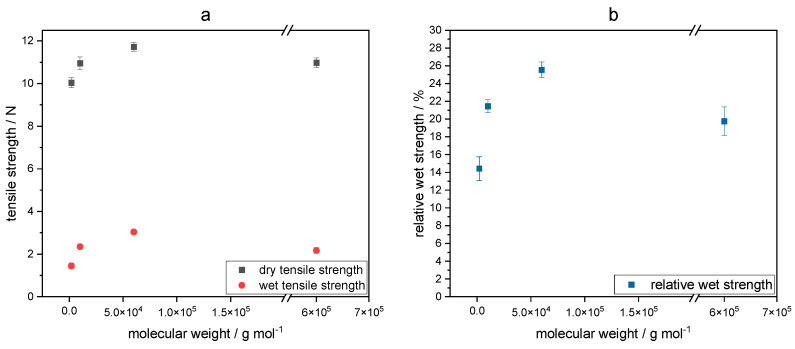
Influence of undegraded keto-HPC cross-linked with PEI with different molecular weights on the dry and wet tensile strength (**a**) and relative wet strength (**b**) in paper. The coating weight of keto-HPC + PEI is 1.5 wt% related to dry fibers. Note that the degree of branching of these investigated PEIs remains the same but polydispersity differs [43].

However, the dry tensile strength also slightly increases. These results suggest that the molecular weight has a stronger impact on the wet strength than on the dry strength (see also the equation in Section 4.11). Here, keto-HPC is able to increase the area of contact between cellulose fibers even at low molecular weight, resulting in only a slight decrease in dry strength. Low-molecular-weight keto-HPC is much less likely to have interfiber bonding between cellulose fibers, resulting in a loss of wet strength [5]. At a molecular weight <1.5 × 10^5^ g mol^−1^, the wet strength results achieved are only slightly higher than the PEI reference. Therefore, its assumed that at this degradation level (and molecular weight), keto-HPC only has a minor impact on improving wet strength. If undegraded keto-HPC is cross-linked with PEI of a different molecular weight, the wet strength in paper is influenced in a similar manner, as shown in Figure 9. Here, different commercially available PEI samples (abbreviated to PEI I–IV in the material Section 4.1) in the molecular weight range of 2.0 to 600 × 10^3^ g mol^−1^ were chosen to study the influence on wet strength in paper. Note that the PEI samples I–IV have the similar degree of branching characterized by Harpe and coworkers but differ in polydispersity [43].

### 2.3. Polymer Distribution of Cross-Linked Keto-HPC in Paper

We assume that the molecular weight of the wet strength system made from keto-HPC/polyamine influences the polymer distribution in paper, resulting in wet strength changes. Thereby, we hypothesized that high-molecular-weight keto-HPC accumulates on fiber–fiber intersections during drying, resulting in high-wet-strength papers. For this purpose, we investigated lab-made paper samples strengthened with keto-HPC/PEI by fluorescent confocal laser scanning microscopy (CLSM) to study the polymer distribution of undegraded, fluorescent-labeled keto-HPC in paper and gain a better understanding of the strengthening mechanism. For fluorescent labeling of keto-HPC, ethylamine rhodamine B (0.005 eq. related to carbonyl functions of keto-HPC) was covalently bonded to keto-HPC, similarly to the reaction with polyamines. In the next step, lab-made paper samples of cotton linters were coated via the previous mentioned procedure (experimental Section 4.6) with labeled keto-HPC and PEI to result in a coating weight of 1.5 wt% based on dry fibers.

**Figure 10:**
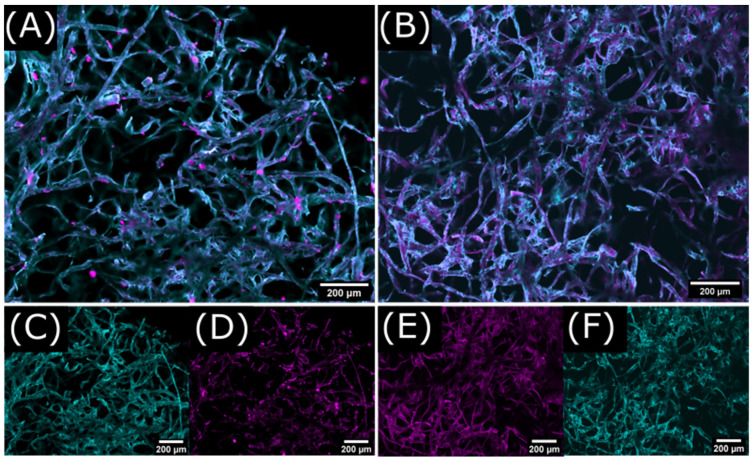
CLSM images with merged rhodamine B and CFW channels for comparison of undegraded keto-HPC (**A**) and strongly degraded keto-HPC (**B**) in lab-made cotton linter paper, both cross-linked with PEI. The separation of rhodamine B ((**C**) resp. (**E**)) and CFW channel ((**D**) resp. (**F**)) allows for a better distinction of labeled keto-HPC and stained cellulose fibers.

To distinguish labeled keto-HPC from cellulose fibers during the CLSM acquisition, fibers were stained with calcofluor white (CFW) prior to keto-HPC application (see experimental Section 4.10). This dye is known to bind to the cellulose surface and allows for better visibility of the fiber network and better distinction from labeled keto-HPC during fluorescence microscopy. The merged channels of CFW and rhodamine B reveal that undegraded, labeled keto-HPC cross-linked with PEI accumulates between fiber–fiber intersections and directly on the fiber surface (Figure 10A). The separated channels of CFW and rhodamine B are also shown in Figure 10C,D, allowing for a better distinction of labeled keto-HPC and stained cellulose fibers.

**Figure 11:**
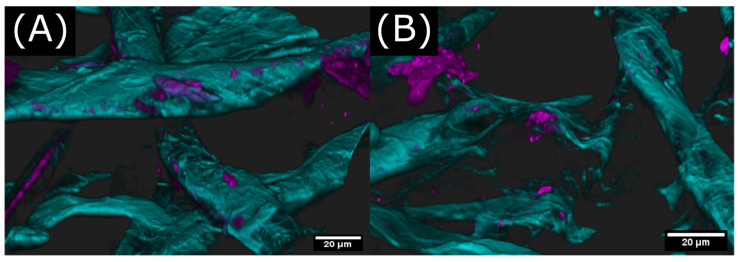
Magnified CLSM image via z-stack acquisition: Here, labeled keto-HPC accumulates on the fiber surface and also penetrates through the fiber wall (**A**); in addition, polymer accumulations of keto-HPC are located within fiber–fiber intersections and welding fibers together (**B**).

The comparison of the CLSM images in Figure 10 of undegraded and degraded keto-HPC allows for a deeper insight on paper wet strength development and its influence by the molecular weight of the used macromolecules. If keto-HPC is degraded (i.e., the molecular weight is lower) prior to its application in paper, the final polymer distribution in paper changes significantly. We observed a significant reduction in polymer accumulations compared to undegraded, i.e., high-molecular-weight keto-HPC. Note that keto-HPC still covers the fiber surface (Figure 10E). At a closer look, magnified polymer accumulations wield cellulose fibers together (Figure 11B). This also happens on a larger scale, as shown in Figure S6. Nevertheless, a considerable amount of keto-HPC is not only located at the fiber–fiber intersections but directly on the fiber far away from fiber–fiber crossing points (Figure 10E). If the fiber is covered by a polymer gel consisting of cross-linked keto-HPC, the latter can prevent fiber swelling, and thus stiffens the fiber, which as a result can improve the wet strength, as shown recently [17]. However, the increase in wet strength is not due to hydrophobization of the cellulose fibers, as dynamic imbibition studies of a water drop as reference suggest (see Figures S7 and S8). In addition, keto-HPC penetrates the fiber walls in some cases, as can be inferred from Figure 11A. At this point, we thus assume that the wet-strengthening effect of keto-HPC is influenced by two major contributions: the reinforcement of the fiber–fiber intersection through polymer accumulation and gelation (by cross-linking), and secondly, the prevention of fiber swelling by a polymer coating of cross-linked keto-HPC gel. Therefore, the low molecular weight of keto-HPC leads to a reduction in the cross-linking possibility of keto-HPC and polyamine during the drying process. As a result, a polymer gel is created that does not sufficiently bind the fibers together due to reduced cross-linking points between fibers.

## 3. Conclusions

In this work, we show that it is possible to use a combination of different polymers of biogenic origin as sustainable and nontoxic cross-linkers within paper to enhance the mechanical strength in wet environments. The system investigated was based on oxidized hydroxypropyl cellulose (keto-HPC). In combination with different polymeric amines (chitosan, polyethylenimine and aminated Kraft lignin, respectively), we show that the formation of thermal cross-links of the respective polymers absorbed to the surface of paper fibers can lead to a strong increase in the relative wet strength of the paper sheet. The keto-HPC/polyamine system is comparable to well-established wet strength agents such as PAAE in terms of wet strength efficiency, while at the same time avoiding AOX contamination. A remarkably high relative wet strength in paper of almost 15% can be achieved by using only 0.75 wt% of keto-HPC + polyamine (relative to the mass of dry fibers).

In a systematic study, we further observed a strong influence of the molecular weight of the used reactive polymeric precursors on the resulting wet strength, and thus, the latter parameter can be easily used to fine-tune the mechanical properties of polymer-cross-linked paper materials in the wet state. A closer look of the polymer gel distribution inside the paper by means of CLSM reveals that polymer accumulations wielding fibers together and that keto-HPC is also capable of penetrating the fiber wall. Although the exact physical cause for this finding is not fully understood, we assume that keto-HPC contributes towards wet strength by reinforcing fiber–fiber intersections through polymer accumulations and by preventing fiber swelling through a polymer coating of cross-linked keto-HPC gel. If keto-HPC is strongly degraded to a low molecular weight by ultrasonic treatment, the polymer distribution of keto-HPC in paper changes by means of fewer polymer accumulations, and wet strength decreases significantly. This insight into the wet strength mechanisms of the keto-HPC/polyamine system could lead to new approaches for the development of alternative wet strength agents in the future.

## 4. Materials and Methods

### 4.1. Materials

Hydroxypropyl cellulose (Klucel G Industrial Grade, M_w_ = 9.6 × 10^5^ g mol^−1^ (determined by SEC), MS = 5.83 (see determination, Supplementary Materials Section S4), degree of substitution (hydroxypropyl) 2.2 (manufacturer details), 2.2.6.6-tetramethyl-piperidinyl-oxyl (Sigma-Aldrich, St. Louis, MO, USA, 98%), sodium bromide (Merck KGaA, Darmstadt, Germany, 99%), sodium hypochlorite (VWR, 14% Cl_2_ in aqueous solution), sodium sulfate (Grüssing, Filsum, Germany, 99%), branched PEI I (Sigma-Aldrich, 50% aqueous solution, M_w_ = 750 × 10^5^ g mol^−1^, M_n_ = 60 × 10^5^ g mol^−1^ (manufacturer details, no method disclosed), degree of branching (DB) = 1.13; PEI II (Sigma-Aldrich, 50% aqueous solution, M_r_ = 600–1000 × 10^5^ g mol^−1^, (manufacturer details, no method disclosed), DB = 1.13; branched PEI III (Sigma-Aldrich, M_w_ = 25 × 10^5^ g mol^−1^ by light scattering, M_n_ = 10 × 10^5^ g mol^−1^ by SEC (manufacturer details), DB = 1.30 branched PEI IV (Sigma-Aldrich, M_w_ = 2 × 10^3^ g mol^−1^ by light scattering, M_n_ = 1.60 × 10^5^ g mol^−1^ by SEC (manufacturer details), DB = 1.13; chitosan (Glentham Life Sciences, Corsham, UK, 5 CP, M_w_ = 7.8 × 10^4^ g mol^−1^, M_n_ = 2.4 × 10^4^ g mol^−1^ by SEC, degree of deacetylation (DD) = 90%) were used as received. All deuterated solvents for NMR analysis were purchased from Sigma-Aldrich.

### 4.2. Synthesis of Keto-HPC with DOx 1.5 via TEMPO-Mediated Oxidation

HPC was oxidized as described in our previous work [25]. In a typical procedure, a solution made from 5 g of HPC (10.0 mmol, 1 eq.) dissolved in 161.7 mL of deionized water was added in a 500 mL round flask. To this HPC solution, 230 mg of sodium bromide (2.24 mmol, 0.224 eq.) was added, and the reaction mixture was cooled down with an ice bath. Before starting the reaction by the adding of sodium hypochlorite, a fresh solution of TEMPO (174 mg (1.11 mmol, 0.111 eq.) in 15 mL deionized water) was made and added to the reaction mixture. Prior to the addition of sodium hypochlorite, the concentration of sodium hypochlorite was determined via iodometric titration (see Supplementary Materials, Section S5). To start the oxidation reaction, 10.9 mL aqueous sodium hypochlorite solution (21.12 mmol, 2.11 eq.) was added dropwise within 15 min. After an additional 15° min of stirring, the reaction mixture was slowly added to 700 mL of a saturated sodium sulfate solution. This mixture was stirred for an additional 30 min and the precipitated product was collected via centrifugation. The collected product was dissolved in deionized water and dried via lyophilization for several days to yield a white product. The reaction product is stored in the freezer until further usage. For the determination of DOx, ^1^H- NMR via 300 MHz Advance III NMR from Bruker was used (see Figure S1 in Supplementary Materials). Furthermore, NMR results were validated by redox titration of a keto-HPC solution with hydroxylamine hydrochloride acid in our previous work [25]. Both methods yield similar results.

### 4.3. Hydrogel Formation Made from Keto-HPC Cross-Linked with Branched PEI

A total of 40 mg keto-HPC DOx1.5 was dissolved in 3.96 g distilled water to result in a 4 wt% solution. To this solution was added 0.583 mL PEI solution with concentration of 10 mg mL^−1^. The pH was adjusted to 7 via the addition of 1 M HCl. The resulting solution was stirred for 1 h with the help of a magnetic stirrer and freeze-dried overnight.

### 4.4. Hydrogel Formation Made from Keto-HPC Cross-Linked with KL-DETA

A total of 40 mg keto-HPC DOx1.5 was dissolved in 3.96 g distilled water to result in a 4 wt% solution. To this solution was added 150 mg KL-DETA. The resulting solution was stirred for 1 h with the help of a magnetic stirrer and freeze-dried overnight.

### 4.5. Hydrogel Formation Made from Keto-HPC Cross-linked with Chitosan

A total of 160 mg keto-HPC DOx1.5 was dissolved in 3.84 g distilled water to result in a 4 wt% solution. To this solution was added 3.86 g a solution of chitosan in 1% acetic acid. The resulting solution was stirred with the help of a magnetic stirrer for 1 h and freeze-dried overnight.

### 4.6. Production of Lab-Made Paper

Cotton linter pulp was refined in a Voith LR lab refiner with an effective specific energy of 200 kWh t^−1^ and a pulp concentration of 4.0 wt%. With the help of the Rapid-Koethen hand sheet maker, a series of paper sheets with a grammage of 100 g m^−2^ was made according to DIN 54358 and ISO 5269/2 and was stored in norm climate for at least 24 h. Lab-made paper sheets containing PAAE were made with the same procedure as described in Supplementary Materials, Section S6.

### 4.7. Application of Keto-HPC and Amine Cross-Linker to Paper via Size Press—General Procedure

Lab-made paper with a grammage of 100 g m^−2^ was impregnated with a solution of keto-HPC and amine cross-linker via size press with an applied pressure of 0.5 bar and a controlled coating speed of 1 m min^−1^. Keto-HPC and amine cross-linkers were dissolved separately in Mili-Q^®^ water, and defined aliquots of these polymeric solutions were mixed to achieve total polymer concentrations of 0.5 wt%, 1.5 wt%, 3.0 wt% and 6.0 wt% in the final impregnation solution, respectively (Detailed quantities of keto-HPC and polyamine cross-linker for the preparation of coating solution are summarized in Tables S1–S3 in Supplementary Materials, Section S7). The mixtures were prepared at room temperature (time for mixing 30 s) and were then immediately applied on the paper via size pressing. The applied amount of polymer to paper was calculated via gravimetric measurement before and immediately after the coating process using the polymer concentration of the coating solution. The coated cotton linter papers were dried on a Teflon-grid in norm climate (23 °C, 50% rel. humidity) for at least 18 h. The cross-linking of keto-HPC with polyamines in paper was carried out in the same way as the cross-linking of HPC in bulk, as described earlier, so no additional heat treatment was necessary.

### 4.8. Polymer Degradation of Keto-HPC via Ultrasonic Treatment

Keto-HPC with a DOx of 1.5 was dissolved in Mili-Q^®^ water to result in a 4 wt% solution. A total of 25 g of a fresh keto-HPC solution was added to a 50 mL round glass. For ultrasonic treatment, a *BANDELIN electronic* UW 2070 probe was immersed into the keto-HPC solution for 1.5 cm. Before the ultrasonic treatment, the keto-HPC solution was cooled down with an ice bath for 15 min while stirring with 700 RPM. The cold solution was treated with ultrasound for 0.5 h, 1.0 h, 2 h, 4 h and 8 h, respectively, using the *BANDELIN electronic* UW 2070 in combination with a *BANDELIN Sonoplus* with a pulse of 7 and power of 70 ± 1%. The degraded keto-HPC was dried via lyophilization for several days and stored in the fridge at −18 °C for further analysis and application.

### 4.9. SEC Analysis of Degraded Keto-HPC

For SEC analysis, a PSS-Agilent 1200 with a GRAM linear column set and GRAM guard column provided by Polymer Standard Service (PSS, Mainz) was conducted at 70 °C. As a mobile phase, a solution of dimethyl sulfoxide (DMSO) with a lithium bromide of concentration of 5 g L^−1^ was used with a constant flow rate of 0.5 mL min^−1^. Polymethylmethacrylate (provided by Polymer Standard Service) was used as an internal standard.

### 4.10. Fluorescent Labeling of Keto-HPC and Lab-Made Paper Samples

For fluorescent labeling of keto-HPC, aminoethyl rhodamine B was synthesized according to the literature [44] (see Supplementary Materials, Section S8). For covalent labeling of keto-HPC, 24 g of a 4 wt% keto-HPC DOx 1.5 solution (48 mmol, 1 eq.) was added to a 50 mL round flask. In the next step, 3.1 mg of aminoethyl rhodamine (6.4 µmol, 0.1 mol-% referred to keto-HPC) dissolved in 2 mL dried methanol was added and stirred at room temperature for 18 h. The resulting slightly pink solution was dialyzed versus water for 48 h (molecular weight cutoff = 4.8 kDa). The dialyzed solution was dried via lyophilization for 3 days. Lab-made paper samples made of cotton linters with a grammage of 100 g m^−2^ were stained in a 100 µM aqueous calcofluor white (CFW) solution for 10 min and washed with distilled water for 1 h to remove unbound CFW. In the next step, paper samples were coated with labeled keto-HPC, similar to the conditions described in Section 4.7, and dried at norm climate overnight.

### 4.11. Tensile Measurements

Prior to tensile measurements, the lab-made paper sheets were cut into 120 mm × 15 mm samples and stored in norm climate (23 °C and 50% humidity) for at least 24 h. For tensile measurements, a Zwick Roell Z1.0 was conducted with a constant strain rate of 20 mm min^−1^ according to DIN ISO 1924-2 at norm climate. For determination of tensile strength, an average of five measurements were made. For wet tensile strength measurements, cut paper samples were soaked for 5 min in deionized water (pH = 6.0) and measured on the same Zwick tensile strength machine at norm climate. The parameter “wet tensile strength” corresponds to a width of 15 mm. Relative wet strength is composed by dividing absolute wet strength by dry strength of the same sample calculated by the following equation:

$$\mathrm{relative}\;\mathrm{wet}\;\mathrm{strength}\;=\frac{\mathrm{wet}\;\mathrm{tensile}\;\mathrm{strength}}{\mathrm{dry}\;\mathrm{tensile}\;\mathrm{strength}}\times 100\%$$

## Data Availability

The data that support the findings of this study are available from the corresponding author upon reasonable request.

## Supplementary Materials

The following supporting information can be downloaded at: https://www.mdpi.com/article/10.3390/gels9030206/s1, Figure S1: ^1^H-NMR spectrum of oxidized hydroxypropyl cellulose in D_2_O for determination of degree of oxidation; Figure S2: ATR-IR spectrum of oxidized hydroxypropyl cellulose (black) and a dried solution of oxidized keto-HPC; Figure S3: ^1^H-NMR spectra of keto-HPC DOx 1.5 after 4 h of ultrasonic treatment; Figure S4: ^1^H-NMR spectrum of hydroxypropyl cellulose in DMSO for determination of molar degree of substitution; Figure S5: ^1^H-NMR spectra of aminoethyl Rhodamine B in CDCl_3_; Figure S6: CLSM overview image via z-stack acquisition of keto-HPC cross-linked with PEI in paper; Figure S7: Contact angle measurements of paper from cotton linter fibers; Figure S8: Contact angle measurements of paper made from cotton linter fibers (100 g m^−2^) with a coating of keto-HPC + PEI (1.0 wt% based on dry fibers); Table S1: Added mass of PAAE to dry cotton linter fibers for production of lab-made papers with PAAE; Table S2: Summary of quantities of keto-HPC and PEI for the preparation of coating solution; Table S3: Summary of quantities of keto-HPC and KL-DETA for the preparation of coating solution; Table S4: Summary of quantities of keto-HPC and chitosan for the preparation of coating solution.
