# Supplementary material for: Cross-Linking of Oxidized Hydroxypropyl Cellulose in Paper: Influence of Molecular Weight and Polymer Distribution on Paper Wet Strength Development
Source: Gels. 2023 Mar 9;9(3):206. doi: 10.3390/gels9030206 (PMC10048462; doi:10.3390/gels9030206)
Supplement: Supplementary file 1 [file gels-09-00206-s001.zip › gels-2234649-supplementary.pdf]

Supporting Info

Cross-linking of oxidized hydroxypropyl cellulose in paper: influence of molecular weight and polymer distribution on paper wet strength development

David Seelinger[1] and Markus Biesalski[1,*]

[1] Ernst-Berl-Institut Macromolecular and Paper Chemistry, Technical University Darmstadt; Alarich-Weiss-Str. 8, 64287 Darmstadt, Germany

* Correspondence: markus.biesalski@tu-darmstadt.de; Tel.: +49 06151 1623822

S1. Determination of degree of oxidation of oxidized hydroxypropyl cellulose

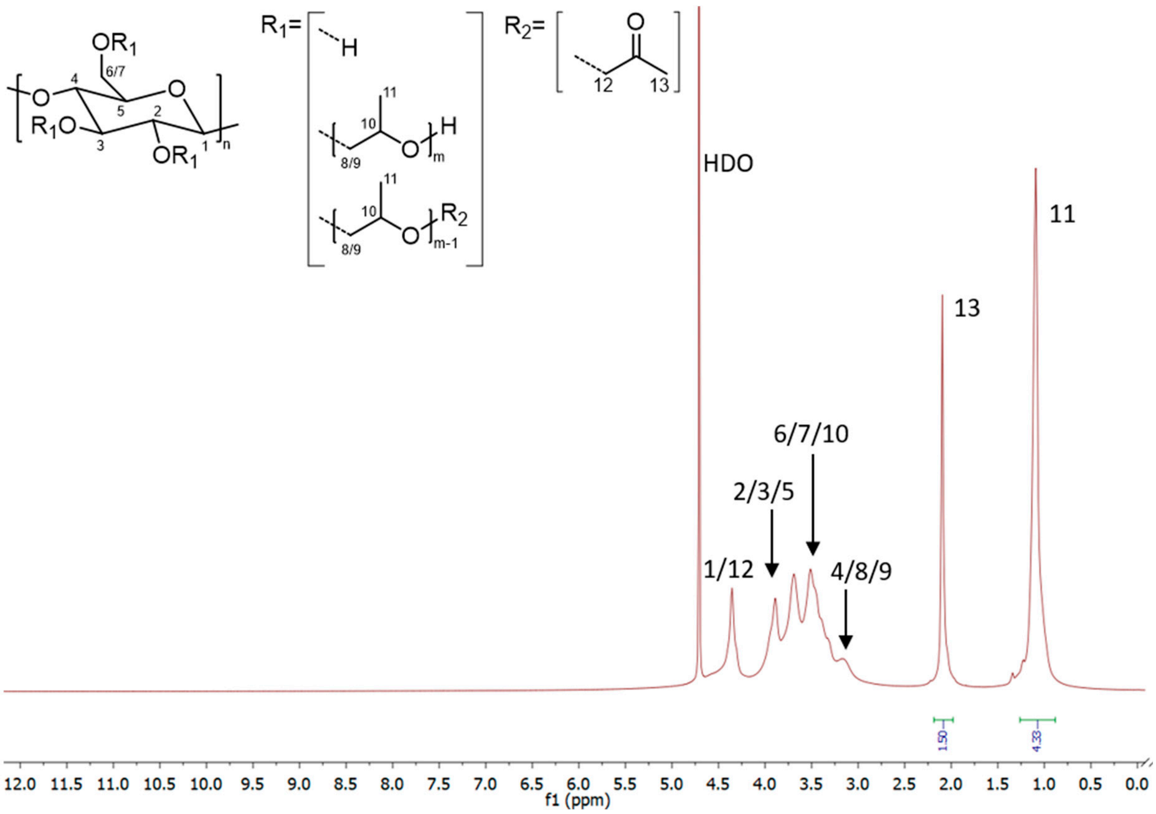

Figure S1. ^{1}H-NMR spectrum of oxidized hydroxypropyl cellulose in D_2O for determination of degree of oxidation.

S2. Determination of degree of oxidation of oxidized hydroxypropyl cellulose

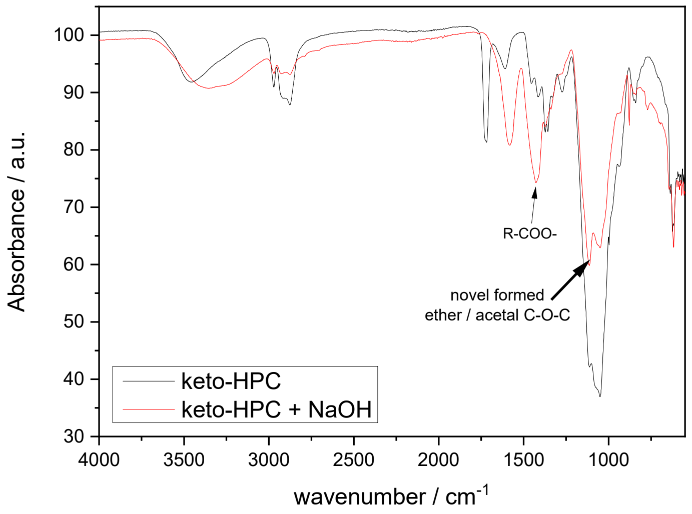

Figure S2. ATR-IR spectrum of oxidized hydroxypropyl cellulose (black) and a dried solution of oxidized keto-HPC.

S3. Investigation of the chemical alternation of keto-HPC after ultrasonic treatment

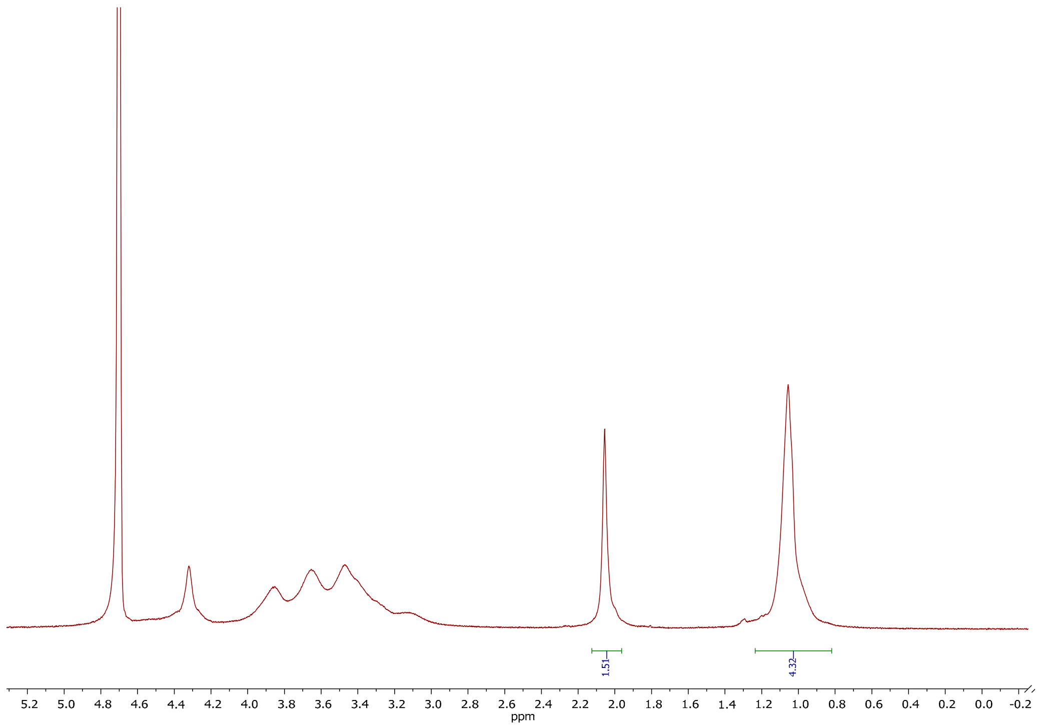

Figure S3. ^{1}H-NMR spectra of keto-HPC DOx1.5 after 4 h of ultrasonic treatment.

S4. Determination of molar degree of hydroxypropyl substitution of HPC

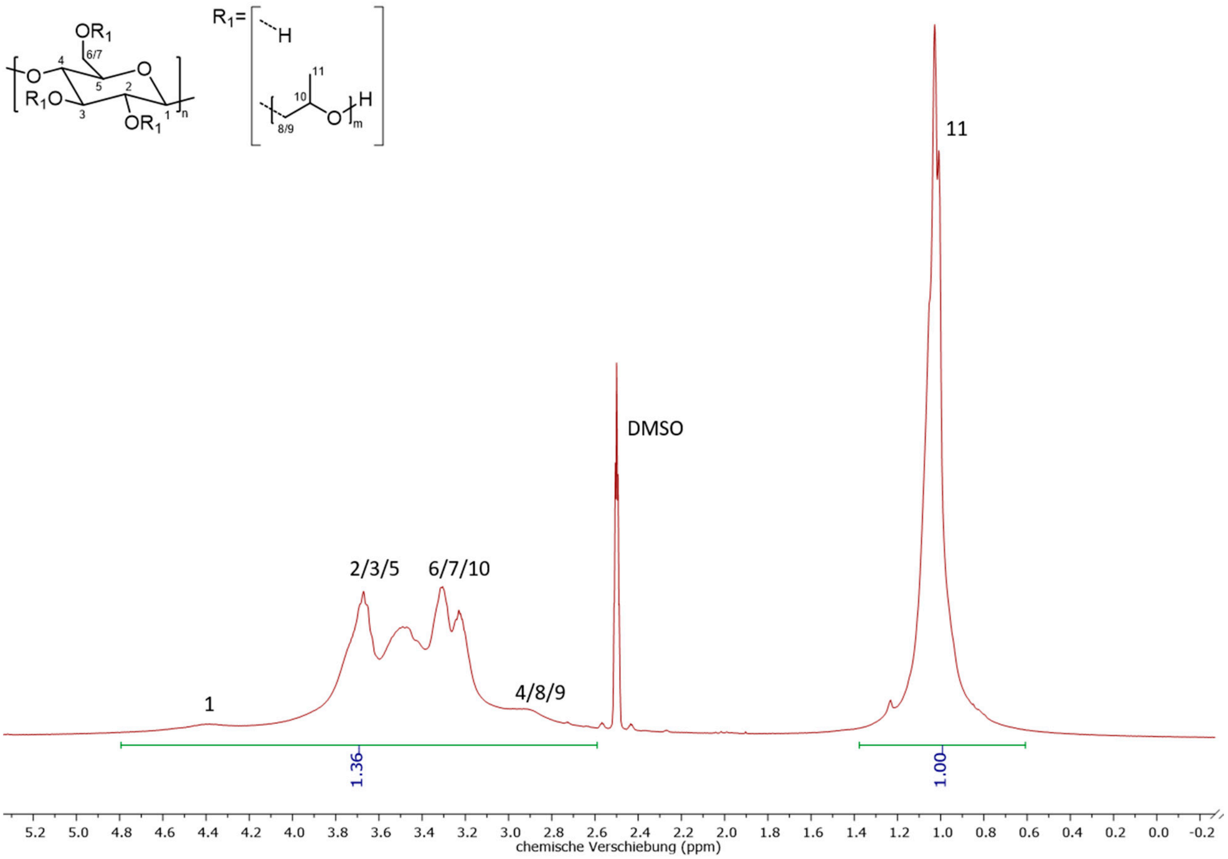

Figure S4. ^{1}H-NMR spectrum of hydroxypropyl cellulose in DMSO for determination of molar degree of substitution.

$$MS = \frac{7 \cdot I_{1.25\,ppm}}{3 \cdot (I_{2.6-4.8\,ppm} - I_{1.25\,ppm}}$$

$$MS = \frac{7 \cdot 1}{3 \cdot (1.36 - 1)} = 5.83$$

S5. Iodometric titration of aqueous sodium hypochlorite solution

A total of 5 mL of sodium hypochlorite stock solution was pipetted into a 100 mL volumetric flask and diluted with water. A total of 10 mL of the resulting solution was pipetted into a 250 mL Erlenmeyer flask and diluted with approximately 80 mL water, then 4 mL 25 % HCl-solution and 3 g potassium iodide were added. After complete dissolving of iodide, the resulting solution was titrated with a 0.5 M solution of sodium thiosulfate pentahydrate until a slight yellowish color appeared. In the next step, 2 mL of a zinc iodide starch solution was added, and the titration continued until the violet-black color disappeared. The titration was repeated twice. (V=19.375 mL)

$$ß_{NaOCl}= \frac{c_{Na_2S_2O_3}*V_{Na_2S_2O_3}*M_{NaOCl}*\text{dilution factor}}{2*V_{NaOCl}} \quad \text{(Eq. S1)}$$

$$ß_{NaOCl} = \frac{0,5\ \frac{mol}{L}*19,375\ mL*74,44\ \frac{g}{mol}*10}{2*25\ mL} = 144,23\ \frac{mg}{mL} \quad \text{(Eq. S2)}$$

S6. Production of lab-made paper with PAAE

A total of 33.0 g of cotton linter fibers were soaked in 3.0 L of distilled water for 48 h. The soaked fibers were suspended in water with the help of the fiber disintegrator for 75.000 rounds. The disintegrated fibers were diluted with 20 L distilled water in a fiber solution suspender. For the formation of lab-made papers, first, ~2000 g of the fiber solution was added in a beaker and then PAAE solution with a concentration of 2.5 wt% was added (see table below). Paper was formed and dried according to the DIN 54358 and ISO 5269/2 and was stored for at least 24 h in norm climate (23 °C, 50 % humidity) until further usage.

Table S1. Added mass of PAAE to dry cotton linter fibers for production of lab-made papers with PAAE.

Concentration of PAAE based on dry fibers [wt%]	added PAAE solution (2.5wt%) [g]
0.2	0.25
0.5	0.63
0.75	0.94
1.0	1.26
2.0	2.51
5.0	6.28

S7. Application of keto-HPC and polyamine solution to cotton linter papers via size press

Table S2. Summary of quantities of keto-HPC and PEI for the preparation of coating solution.

total polymer concentration [wt-%]	keto-HPC [mg]	MiliQ® water for dissolution of keto-HPC [g]	added PEI solution (10 mg/mL) [mL]
0.50	60	12.815	0.875
1.5	180	10.945	2.625
3.0	300	6,78	4.375
6.0	783	8.86	5.25 (20mg/mL)

Table S3. Summary of quantities of keto-HPC and KL-DETA for the preparation of coating solution.

total polymer concentration [wt-%]	keto-HPC [mg]	MiliQ® water for dissolution of keto-HPC [g]	added KL-DETA [mg]
0.5	30	27,625	112.5
1.5	40	11,525	150
3.0	80	10,375	300
6.0	200	10,075	750

Table S4. Summary of quantities of keto-HPC and chitosan for the preparation of coating solution.

total polymer concentration [wt-%]	keto-HPC [mg]	MiliQ® water for dissolution of keto-HPC [g]	added chitosan solution (2 wt-% in 1 wt-% acetic acid [g]
0.5	60	16.26	1.45
1.5	180	13.27	4.35
3.0	400	9.71	9.67
6.0	800	9.31	9.67 (4 wt-%)

S8. Synthesis of aminoethyl Rhodamine B

A solution of 750mg Rhodamine B (1.69 mmol; 1 eq.) in 150 mL ethanol, absolute and 2.10 mL ethylene diamine (50 mmol; 29.6 eq.) in 15 mL ethanol was mixed in a 250 mL flask and refluxed for 48 h (oil bath temperature = 85 °C) until the solution changed color from pink to orange. In the next step, the solvent was removed under lower pressure. After the addition of approximately 25 mL distilled water, the crude product was extracted with 50 mL ethyl acetate four times. The collected organic phase was dried over sodium sulfate and the solvent was removed under lower pressure. The crude product was purified via flash column chromatography (ethyl acetate : methanol = 1:1; Rf-value product: 0.8-0.9) to obtain a slight orange powder. Note that the product exists in the closed and colorless form after and during column chromatography. Yield: 283 mg (34.4 % d.Th.)

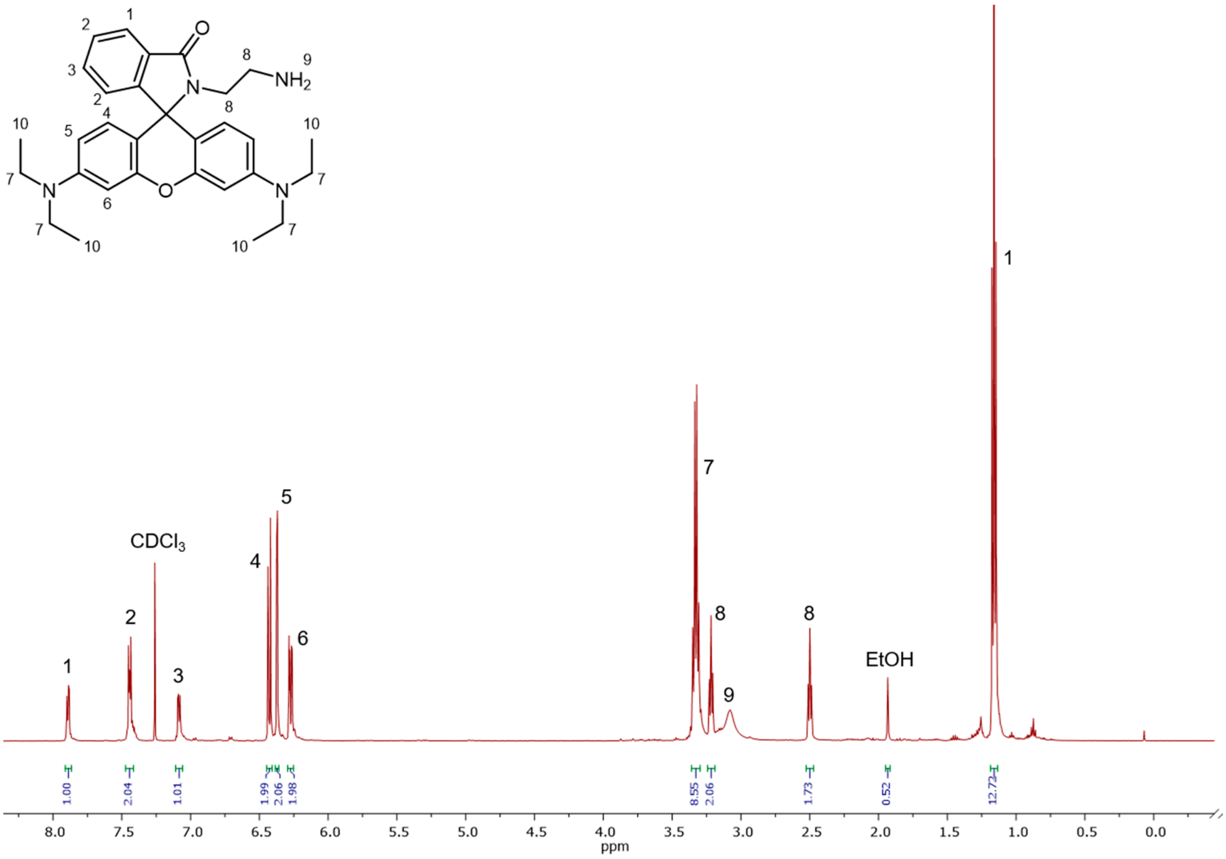

Figure S5. ^{1}H-NMR spectra of aminoethyl Rhodamine B in $CDCl_3$.

S9. Labeling of keto-HPC with ethylamine rhodamine B

A total of 960 mg keto-HPC G DOx1.5 (1.92 mmol, 1 eq.) was added to a 50 mL round flask and dissolved in distilled water to result in a 4.0 wt% solution. In the next step, 4,65 mg aminoethyl rhodamine B (6.40 µmol, 0.005 eq.) dissolved in 2.0 mL dry methanol was added and stirred at 700 rpm at 23 °C for 1 h. The resulting slight pink solution was dialyzed versus distilled water for 48 h and freeze-dried for 3 days to gain a slight pink powder.

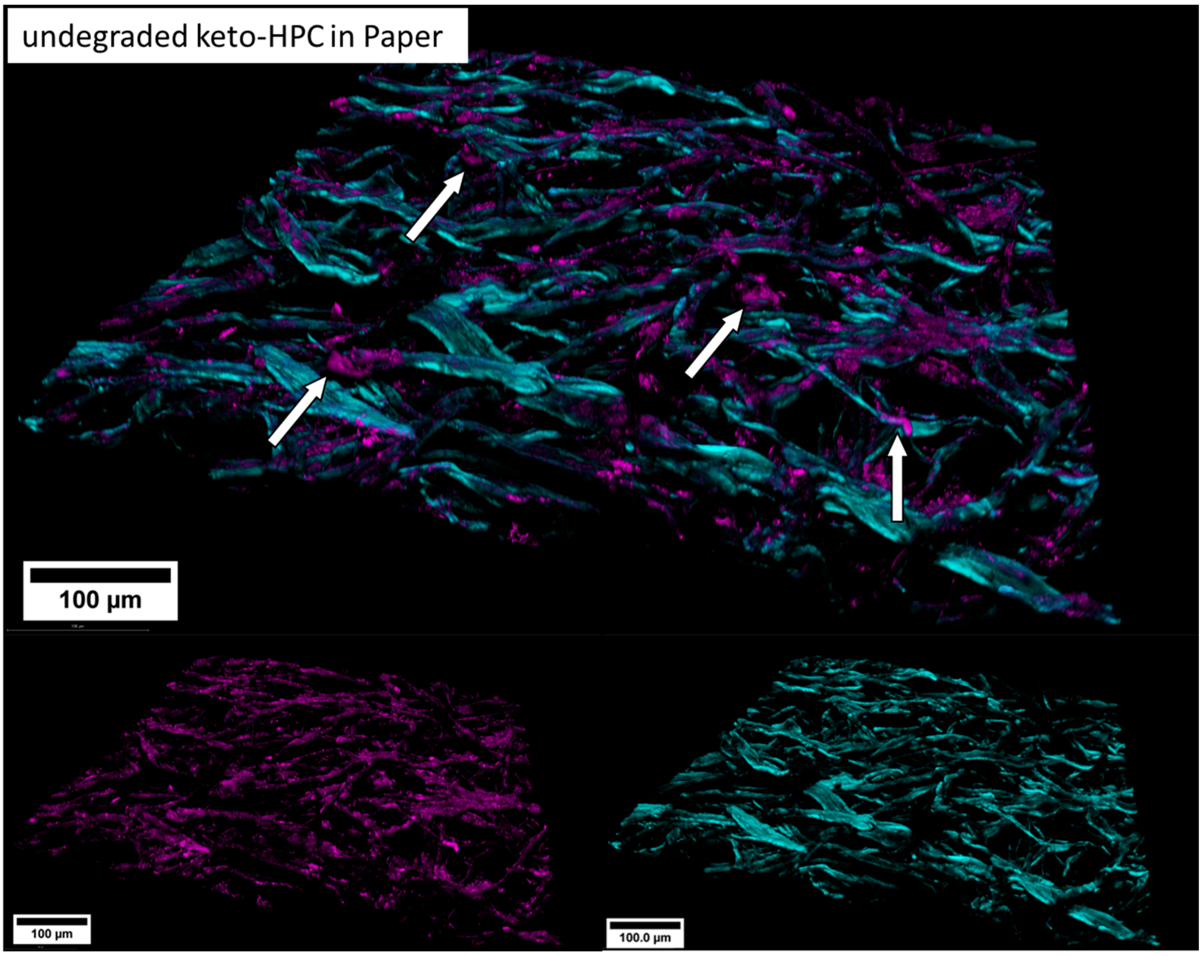

Figure S6. CLSM overview image via z-stack acquisition of keto-HPC cross-linked with PEI in paper.

S10. Contact angle and dynamic imbibition measurements of water on/in paper made from cotton linter fibers with cross-linked keto-HPC/PEI

The contact angle measurements were carried out on a TBU90E device from DataPhysics Instruments GmbH in a norm climate (23 °C, 50% relative humidity). The evaluation took place with the program SCA software. For the measurement of the contact angle, a drop of water (Milli-Q water) with a drop volume of 2 µL and a deposition rate of 0.5 µL s^{-1} suspended from the cannula was used. The substrate was located on a movable stage. In the next step, video capturing via the SCA software was started. The water droplet was deposited on the paper substrate by moving up the stage.

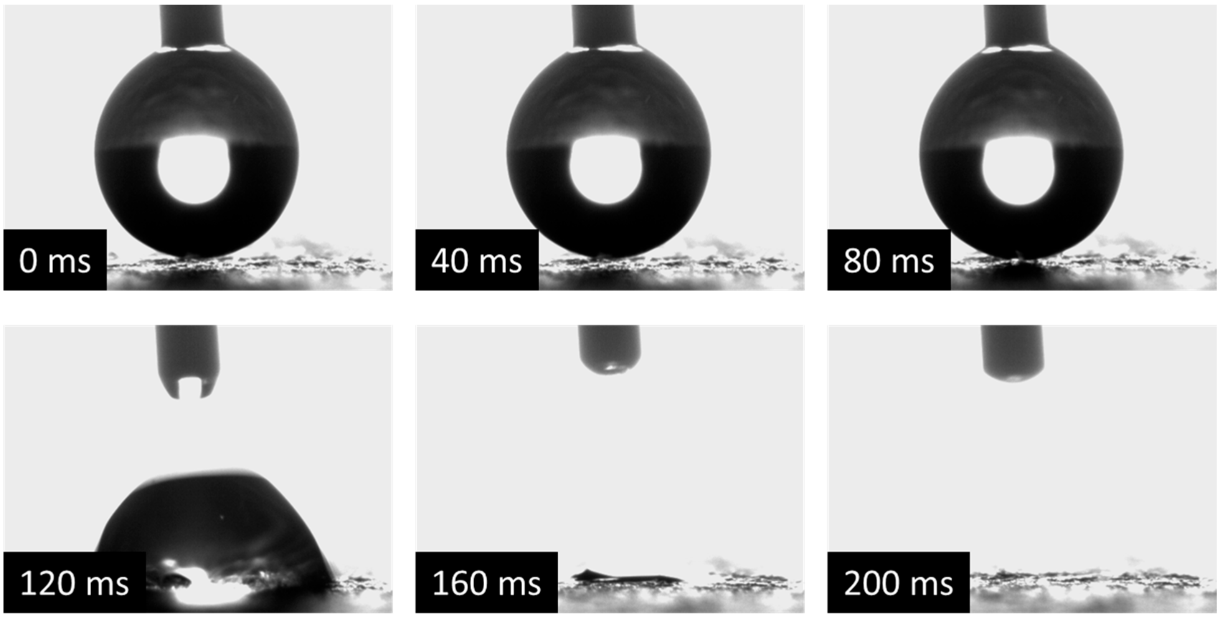

Figure S7. Contact angle measurements of paper from cotton linter fibers.

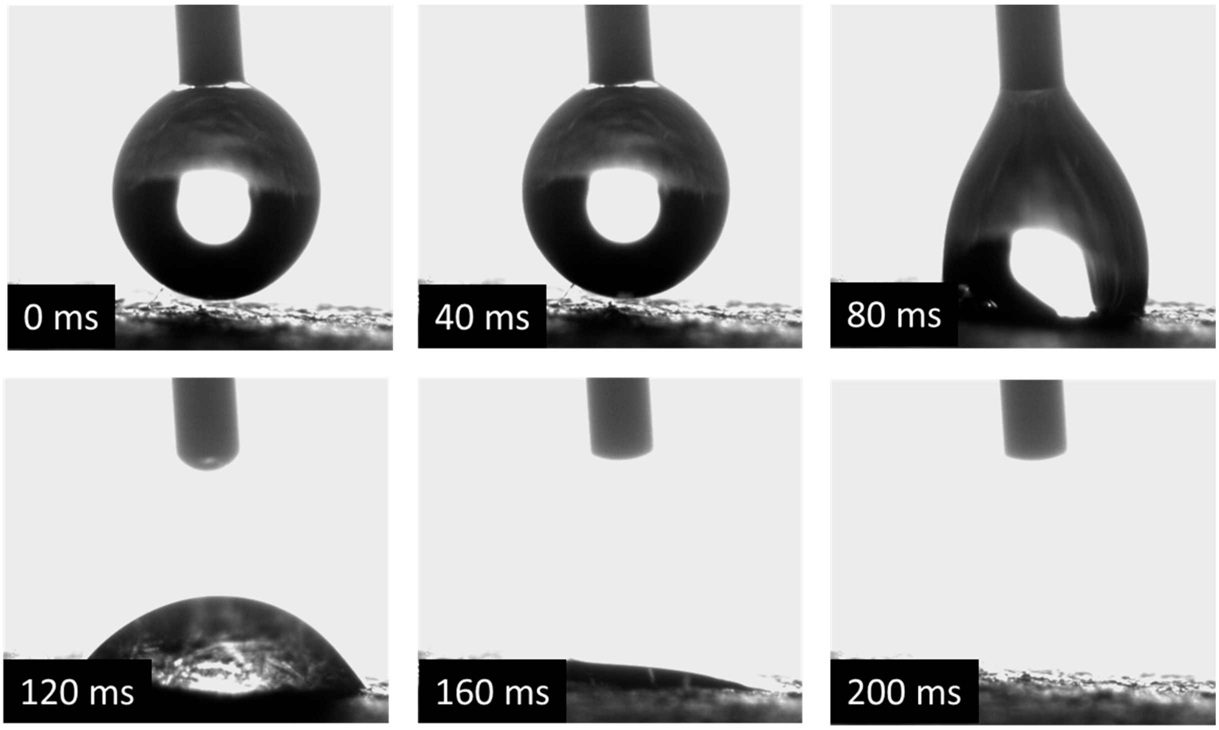

Figure S8. Contact angle measurements of paper made from cotton linter fibers (100 g m^{-2}) with a coating of keto-HPC + PEI (1.0 wt-% based on dry fibers).
